# Magnetism of Otherwise Nonmagnetic Elements: From Clusters to Monolayers

**DOI:** 10.1021/acs.jpcc.4c03592

**Published:** 2024-07-16

**Authors:** Manish Kumar Mohanta, Puru Jena

**Affiliations:** Department of Physics, Virginia Commonwealth University, Richmond, Virginia 23284, United States

## Abstract

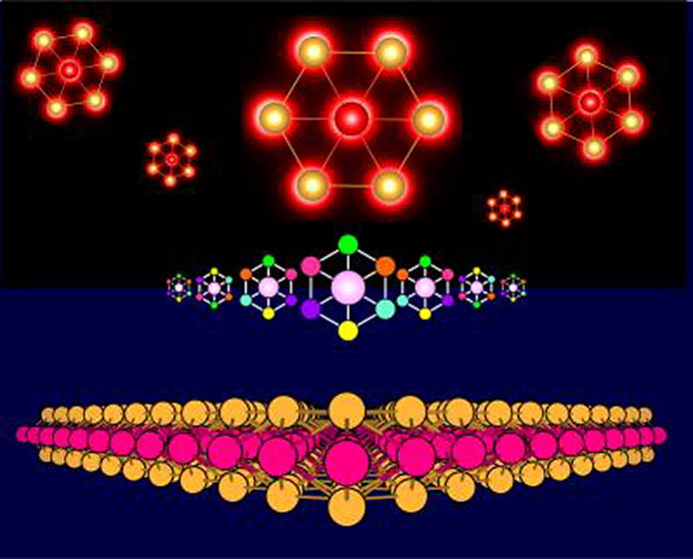

Atomic clusters are known to exhibit properties different from their bulk phase. However, when assembled or supported on substrates, clusters often lose their uniqueness. For example, uranium and coinage metals (Cu, Ag, Au) are nonmagnetic in their bulk. Herein, we show that UX_6_ (X= Cu, Ag, Au) clusters, unlike their nonmagnetic bulk, are not only magnetic but also retain their magnetic character and structure when assembled into a two-dimensional (2D) material. The magnetic moment remains localized at the U site and is found to be 3μ_B_ in clusters and about 2μ_B_ in the 2D structure. In 2D UX_4_ (X = Cu, Ag, Au) monolayers, U atoms are found to be coupled antiferromagnetically through an indirect exchange coupling mediated by the coinage metal atoms. Furthermore, hydrogenation of these monolayers can induce a transition from the antiferromagnetic to the ferromagnetic phase. These results, based on density functional theory, have predictive capability and can motivate experiments.

## 1 Introduction

More than half of the elements in the periodic table possess nonzero spins, yet very few of them remain magnetic in their bulk phases. However, as their size is reduced, some of the clusters of nonmagnetic elements develop finite magnetic moments at the subnano meter length scale due to their large surface-to-volume ratio and reduced coordination number. One of the early observations where topology and symmetry were found to play a role in magnetism was in the Li_4_ cluster which has a spin-singlet ground state and a planar geometry but becomes a spin triplet configuration when it assumes a three-dimensional tetrahedral structure.^1^ This topology-driven magnetic transition was attributed to a delicate balance between Jahn–Teller distortion and Hund’s rule coupling; the former favors a planar structure and zero spin while the latter favors a tetrahedral geometry and spin 1. Subsequently, transition metal atoms such as vanadium (V) and rhodium (Rh) that are nonmagnetic in the bulk were found to be magnetic in clusters.^2−9^ In addition, magnetic moments of transition metal elements get enhanced when their size is reduced to the nanoscale.^10^ While these results are firmly established by theory and experiment in isolated clusters, magnetism of otherwise nonmagnetic elements in the bulk form is rare.

In recent experimental work, Harris et al.^11^ studied the photoelectron spectroscopy of the UAu_6_ cluster, intending to shed light on the UF_6_ cluster which is an important material for the nuclear industry. However, previous experiments were not successful in understanding its electronic properties. As Au behaves like a halogen in small clusters, it was hoped that the study of UAu_6_ could serve as a surrogate for UF_6_. However, unlike the UF_6_ cluster which has zero spin, the UAu_6_ cluster was found to be magnetic with nearly 3μ_B_ magnetic moment localized at the U site.^11^ In addition, UAu_6_ has three nearly degenerate isomers, all of which are magnetic irrespective of their geometry. This raises some fundamental questions. (1) Is it possible that similar clusters containing other coinage metal atoms such as Cu and Ag would become magnetic? Note that due to the relativistic effects, the properties of Au are different from those of Cu and Ag, including their color. (2) Can these clusters retain their geometry and magnetic properties, once assembled to form a crystal? (3) If so, would the coupling be ferromagnetic or antiferromagnetic? (4) Can magnetic coupling be altered by hydrogenation and/or biaxial strain? (5) What type of magnetic exchange interaction between localized spins dominates in these 2D structures?

In this work, we address the above questions. We show that UX_6_ (X = Cu, Ag) clusters, analogous to the UAu_6_ cluster, are magnetic with a magnetic moment of 3μ_B_ localized at the U-site. The geometries as well as magnetism of the UX_6_ (X = Cu, Ag, Au) clusters are retained, once they are assembled into a two-dimensional (2D) structure. The resulting UX_4_ (X = Cu, Ag, Au) monolayers are antiferromagnetic but become ferromagnetic when hydrogenated. These results are based on density functional theory which has been found to correctly account for the photoelectron spectroscopy (PES) experiments of isolated UAu_6_ clusters.^11^

## 2 Computational Details

The ground-state properties of isolated UX_6_ (X = Cu, Ag, Au) clusters are calculated using spin unrestricted density functional theory (DFT) employed in the Gaussian16 package.^12^ The exchange-correlation potential is treated using a Becke 3-parameter Lee–Yang–Parr (B3LYP) hybrid functional.^13,14^ The basis sets used for U and X (Cu, Ag, Au) atoms are correlation-consistent polarized valence double-ξ (cc-pVDZ-PP).^15,16^ The scalar relativistic effects of the core electrons for U and X atoms are incorporated using 60-electron Stuttgart/Cologne energy-consistent effective core potentials (ECP60MDF).^17,18^ Quadratic convergence algorithms are used during the optimization process without any symmetry constraints. The projected density of states, spin density, and photoelectron spectra of the clusters are calculated using the Multiwfn package.^19^ The projected density of states (PDOS) is plotted using a Gaussian broadening function (full width at half-maximum, FWHM = 0.2 eV).

The ground state properties of 2D structures assembled from these clusters are calculated using the DFT incorporated in the Vienna ab initio Simulation Package (VASP).^20,21^ The ion-electron interaction and the exchange-correlation potential are described by using the projector augmented wave (PAW)^22^ method and the generalized gradient approximation due to Perdew–Burke–Ernzerhof (GGA-PBE),^23^ respectively. The effect of spin–orbit coupling (SOC) on the calculated magnetic properties is also taken into account. A kinetic energy cutoff of 500 eV is used. The first Brillouin zone is sampled by a Γ- centered 14 × 14 × 1 k-mesh for the unit cell and 10 × 10 × 1 for the supercell. The GGA+U method^24^ is adopted with an effective Hubbard *U*_eff_ value of 4 eV applied to the 5f orbitals of the uranium atom. We have also performed energy calculations with meta-GGA (SCAN) and *U*_eff_ value of 3 eV. The corresponding results are mentioned in the respective sections. A vacuum layer of 20 Å is added in the *z*-direction to avoid periodic interactions. The convergence criteria for the energy and force of each atom are set to 10^–6^ eV and 0.02 eV/Å, respectively. The PHONOPY code^25^ is used to obtain the phonon dispersion curve via the finite displacement method. The density of states (DOS) plots are analyzed using the VASPKIT^26^ program. To study the interaction of hydrogen and Ag/Au surfaces at a higher temperature, ab initio molecular dynamics simulations (AIMD) are performed at 300 K for 2 ps using the Nosé–Hoover thermostat. The VMD,^27^ Avogadro,^28^ and VESTA^29^ software are used to create the figures.

## 3 Results and Discussion

In the following, we first discuss the geometry, electronic, and magnetic properties of isolated UX_6_ (X = Cu, Ag, Au) clusters. Next, the stability and geometrical properties of 2D structures created by assembling these clusters are discussed. The magnetic properties of the 2D monolayers have been studied to correlate with the results obtained for isolated magnetic clusters. Further, the effect of hydrogenation on magnetic coupling is studied.

### 3.1 Ground-State Properties of UX_6_ (X = Cu, Ag, Au) Clusters

**Figure 1:**
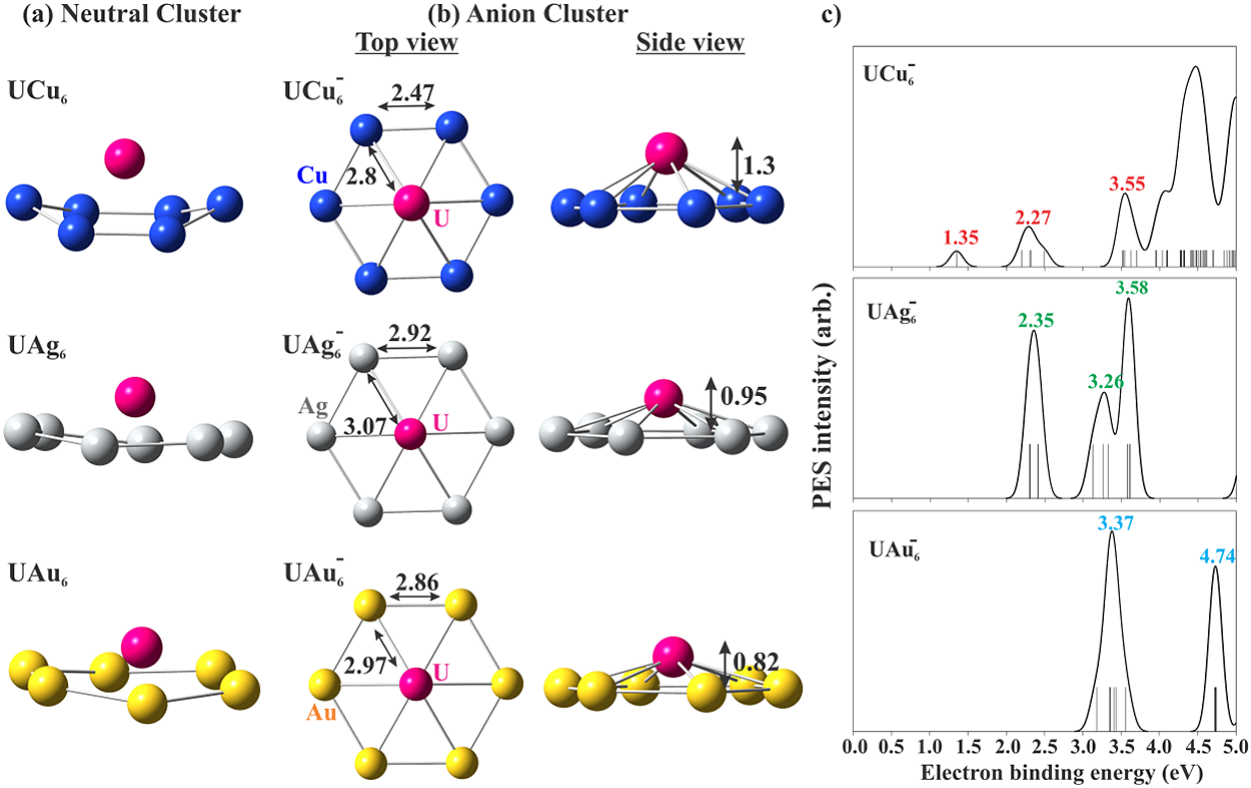
(a) Geometry of neutral UX_6_ clusters (X = Cu, Ag, Au), (b) top and side view of the anionic UX_6_^–^ clusters; distance is in Å, the vertical distance between the U atom and the hexagonal plane is indicated in the figure, (c) calculated photoelectron spectra of UX_6_^–^ clusters.

In a recent joint experimental and theoretical work, Harris et al.^11^ reported three stable nearly degenerate isomers of UAu_6_ clusters among which the properties of the ring-like quasi-two-dimensional structure (six Au atoms forming a planar hexagonal ring with the U atom situated slightly above the center of the hexagon) was found to be consistent with experiment. We have used this geometry as a reference for the study of its iso-structural and iso-electronic UCu_6_ and UAg_6_ cousins. The optimized geometries of the neutral UX_6_ (X = Cu, Ag, Au) clusters with top and side views are presented in Figure 1a. The dynamical stability of these clusters has been confirmed by the calculated positive vibrational frequencies. All six coinage metal atoms form a planar ring with the uranium atom located slightly above the center of the plane. The distances between X-X and X-U in the anionic clusters and the height of the U atom from the X_6_ plane are indicated in Figure 1b.

The ground states of the UX_6_^–^ anionic clusters have quartet spin multiplicity with a magnetic moment of 3μ_B_ localized at the U site. The ground state of the UX_6_ neutral clusters, on the other hand, is a spin quintet with a magnetic moment of 3μ_B_ localized at the U site and 1μ_B_ distributed over the X atoms. In Table S1 of the Supporting Information (SI), we have provided the total energies of the spin singlet, triplet, and quintet state of UX_6_ neutral and spin doublet and quartet state of the UX_6_^–^ anion clusters. From the total energy values, the ground state of neutral and anionic clusters is found to be in quintet and quartet spin states, respectively. The electron affinities (EAs), defined as the energy difference between the ground vibronic states of the anion and the neutral, and the vertical detachment energy (VDE), defined as the energy difference between the anion and the neutral at the anion geometry, are given in Table 1. For reference, the values of calculated EA and VDE of the UAu_6_ cluster in this work are in agreement with the reported experimental value.^11^

**Table 1:** Calculated Adiabatic Electron Affinity (EA) and Vertical Detachment Energy (VDE) of UX_6_^–^ Clusters (X = Cu, Ag, Au)

clusters	EA (eV)		VDE (eV)	
	experiment	theory	experiment	theory
UCu_6_^–^		1.29		1.35
UAg_6_^–^		2.21		2.31
UAu_6_^–^	3.05 ± 0.05	3.07	3.28 ± 0.05	3.18

Next, the photoelectron spectra (PES) of UX_6_^–^ clusters are theoretically simulated based on the generalized Koopman’s theorem. The results are plotted in Figure 1c. The position of the first peak in the PES spectra is equivalent to the VDE of the system, as listed in Table 1. To check the accuracy, we note that the simulated first two peaks of UAu_6_^–^ located at 3.37 and 4.74 eV are in excellent agreement with the experimental photoelectron spectra. The location of the photoelectron intensity peaks for other anion clusters is indicated in Figure 1c.

**Figure 2:**
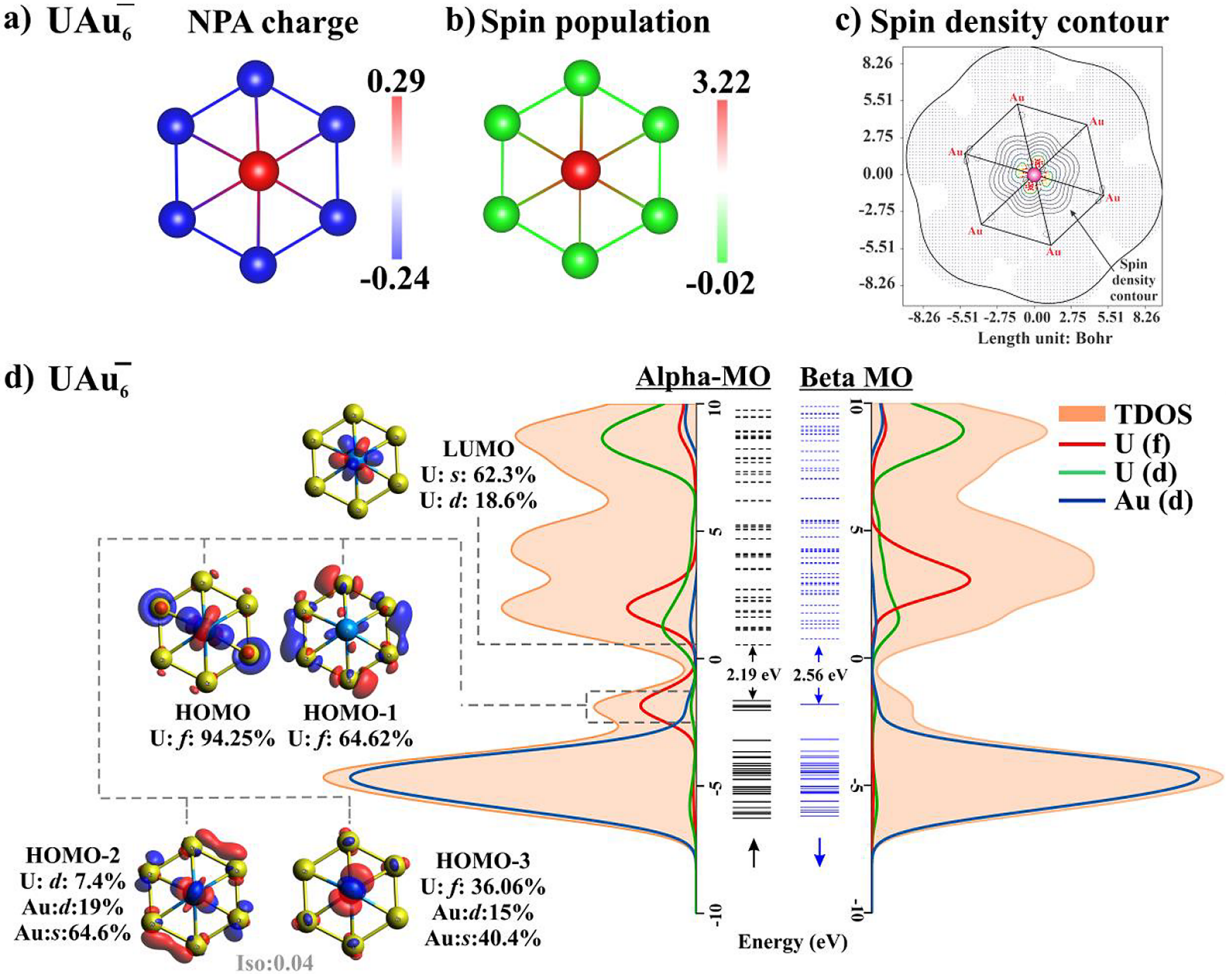
(a) Atomic charge distribution based on natural population analysis (NPA) of UAu_6_^–^, (b) spin population and magnetism analysis, (c) contour plot of the spin density on the XY-plane, (d) electronic structure analysis: partial density of states (pDOS) and selected α-orbitals of UAu_6_^–^; solid/dashed lines indicate occupied/unoccupied molecular orbital.

Figure 2a,b depicts the natural population analysis (NPA) and spin population for UAu_6_^–^. The values of NPA charge and spin populations are listed in Table S2. Furthermore, Figure 2b and data in Table S2 indicate that spin-up electron populations are mainly localized on the uranium atom which is further confirmed by the spin-density contour plot in Figure 2c, whereas the Au-atom possesses a negligible spin population. The electron spin density distribution, calculated by using eq 1, is given as a contour plot on the XY-plane which confirms finite magnetization in the system originating from the localized spin at the U atom site. Here, α/β represents spin-up/spin-down which is also referred to as majority/minority electron spin.
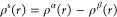
1

From the electronic configuration of uranium and gold atoms (U: [Rn] 5f^3^ 6d^1^ 7s^2^; Au: [Xe] 6s^1^ 4f^14^ 5d^10^), it is quite evident that the HOMO and LUMO will be contributed by the s, d, and f orbitals of the uranium atom and the s/d-orbital of the Au-atom. The density of states (DOS), partial DOS (pDOS) and selected frontier orbitals of UAu_6_^–^ are plotted in Figure 2d and those for the other two clusters are plotted in Figures S1 and S2. The HOMO–LUMO gaps of the majority spin-bands are found to be in decreasing trend UAu_6_^–^ (2.19 eV) > UAg_6_^–^ (1.71 eV) > UCu_6_^–^ (0.34 eV). Furthermore, the pDOS plot indicates the percentage contribution of different atomic orbitals which are in accordance with the electronic configuration, as mentioned earlier. The orbital/atomic composition of the HOMO and LUMO for the majority and the minority spins are listed in Tables S3 and S4. Since the HOMO–LUMO of UAu_6_^–^ are composed of f-orbitals, the HOMO–LUMO gaps of both majority and minority spins are quite large compared to other systems composed of d-orbitals.^30,31^

### 3.2 Geometry and Stability of Two-Dimensional UX_4_ (X = Cu, Ag, Au) Monolayers

**Figure 3:**
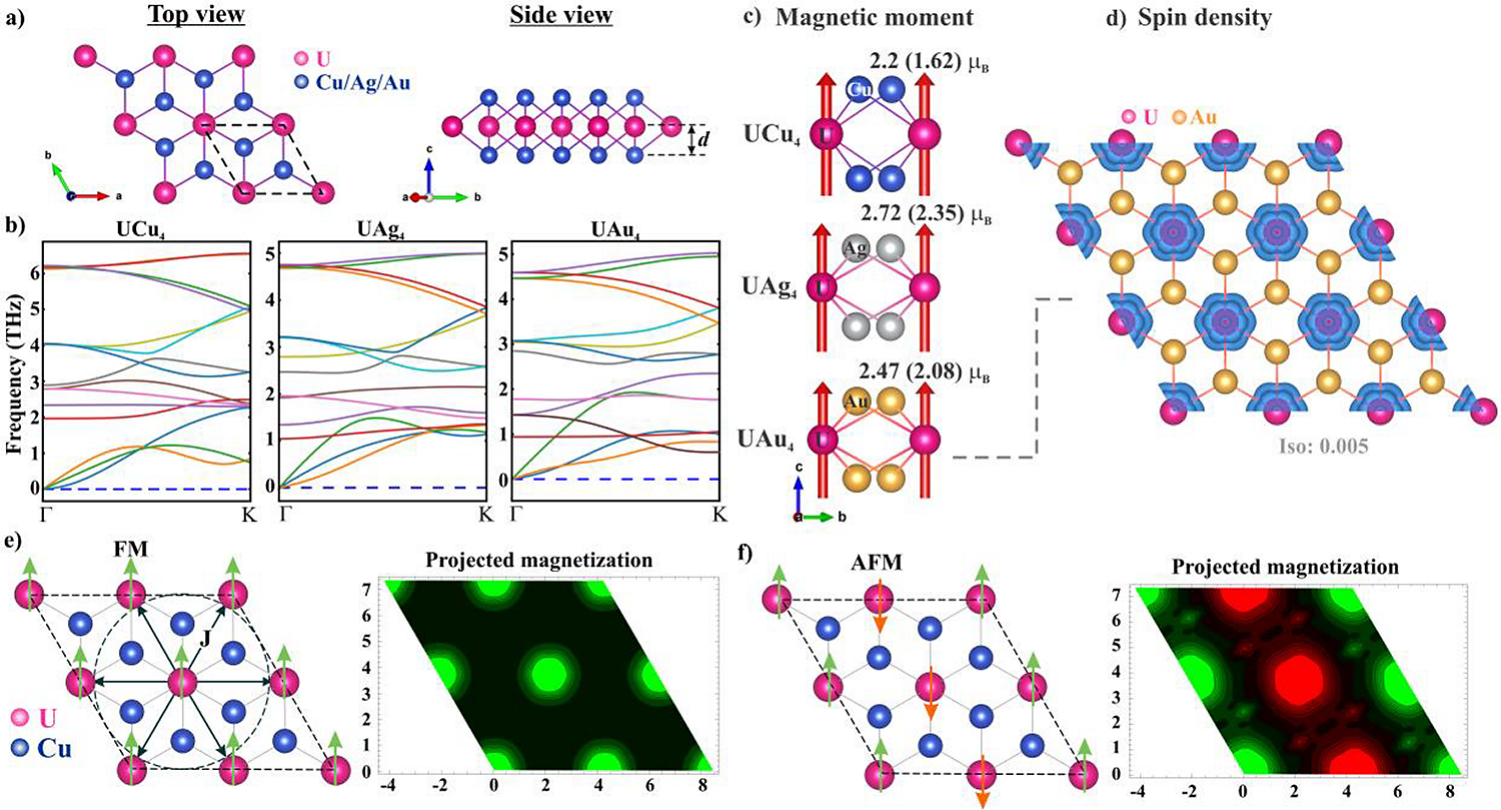
(a) Top and side view of the designed UX_4_ unit cell and their (b) phonon dispersions (colors are random), (c) on-site magnetic moment at the U-site without (with) spin–orbit coupling, (d) spin-density plot of UAu_4_ monolayer, (e,f) top view of FM/AFM state; green/red arrows indicate initialized magnetic moment (±*z*-direction) at the U-site, and projected magnetization of spin density along the *z*-direction; green/red contours indicate localized spin-up/spin-down density, respectively.

To see if these clusters can be used as building blocks of materials where the properties of clusters will remain intact, we constructed three atomic thick hexagonal 2D structures composed of uranium and coinage metal atoms. In monolayer, UX_4_, a triangular network of U atoms is sandwiched between two coinage metal honeycomb lattices as presented in Figure 3a. Each U atom is covalently bonded to 12 X atoms, as shown in Figure S3. The hexagonal 2D crystal structure has a *P*6/*MMM* (*D*_6h_^1^) symmetry group (space group number 191). The optimized geometrical parameters of the UX_4_ monolayers (ML) are listed in Table 2. The bond lengths between U-X in 2D structures follow the same trend as in clusters. Notably, the bond length and the lattice constants of the UAg_4_ monolayer are larger compared to the UAu_4_ monolayer, the difference originating from the larger atomic radius of Ag compared to that of Au. The vertical distances between the U atom and the coinage metal planes, as indicated in Figure 3a, decrease from Cu to Au in the 2D structures. This too is consistent with results from the corresponding clusters. We note that the UAu_2_ crystal^32,33^ has been experimentally synthesized in which the uranium atom is covalently bonded to 12 Au-atoms. The geometrical view is plotted in Figure S3.

**Table 2:** DFT Optimized Geometrical Parameters of the UX_4_ Unit Cell

monolayers	*a* = *b* (Å)	bond length U–X (Å) (2D material)	bond length U–X (Å) (Anionic cluster)	vertical height (*d*) between U and X planes (Å)
UCu_4_	4.23	2.96	2.8	1.66
UAg_4_	4.74	3.15	3.07	1.56
UAu_4_	4.7	3.11	2.97	1.53

Next, the dynamical stability of the designed 2D monolayers has been confirmed from the phonon dispersion plot presented in Figure 3b. Analysis of the phonon spectra shows that all 15 phonon branches (three acoustic and 12 optical) have positive frequencies. A quadratic dispersion of three acoustic branches near the Γ-point is observed which is essential in a dynamically stable structure.^34−36^

### 3.3 Magnetic Properties and Spin Exchange Interaction

The spin-polarized calculations carried out without (with) spin–orbit coupling (SOC) yield an on-site magnetic moment of 2.2μ_B_ (1.62μ_B_), 2.72μ_B_ (2.35μ_B_) and 2.47μ_B_ (2.08μ_B_) in UCu_4_, UAg_4_, and UAu_4_ 2D monolayers. The magnetic moments are localized at the U atom site in the unit cell as can be visualized in Figure 3c. The percentage orbital contribution to magnetic moment is provided in Table S5. The magnetic moment of the uranium atom in the bulk UAu_2_ structure is calculated to be 2.44μ_B_, as indicated in Figure S3. Figure 3d represents the spin density (ρ_↑_–ρ_↓_) of the UAu_4_ ML, suggesting that the magnetism originates entirely from the U atoms whereas the Au atom remains nonmagnetic.

To investigate the spin exchange coupling for 2D monolayers, both ferromagnetic (FM) and antiferromagnetic (AFM) couplings between the nearest-neighbor U atoms are considered. Assuming that the nearest neighbors ⟨*ij*⟩ have the same interaction strength, the magnetic coupling Hamiltonian^37^ can be written as
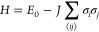
2where σ_*i*_ ∈ {−1, + 1} represents Ising spins, *J* is the exchange energy between the neighboring site indicated in Figure 3e, ⟨*ij*⟩ represents the sum over all nearest neighbors, and *E*_0_ represents the nonmagnetic part of the energy. In the case of a positive exchange integral *J* > 0, the system is in a ferromagnetic ground state such as | ↑ ↑ ↑ ↑ ⟩ or | ↓ ↓ ↓ ↓ ⟩. In contrast, when the exchange integral is negative *J* < 0, nearest-neighbor magnetic moments tend to align antiferromagnetically in the form | ↑ ↓ ↑ ↓ ⟩.^38,39^ A 2 × 2 supercell is constructed from the optimized unit cell to study the preferred magnetic ground state. The magnetic configurations and projected magnetization of the spin density along the *z*-direction for FM and AFM are shown in Figure 3e, f. The total energy of 2 × 2 supercells in their FM and AFM states can be expressed as
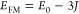
3
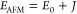
4

The magnetic exchange coupling constants per unit cell can be calculated using the following relation:
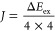
5

**Figure 4:**
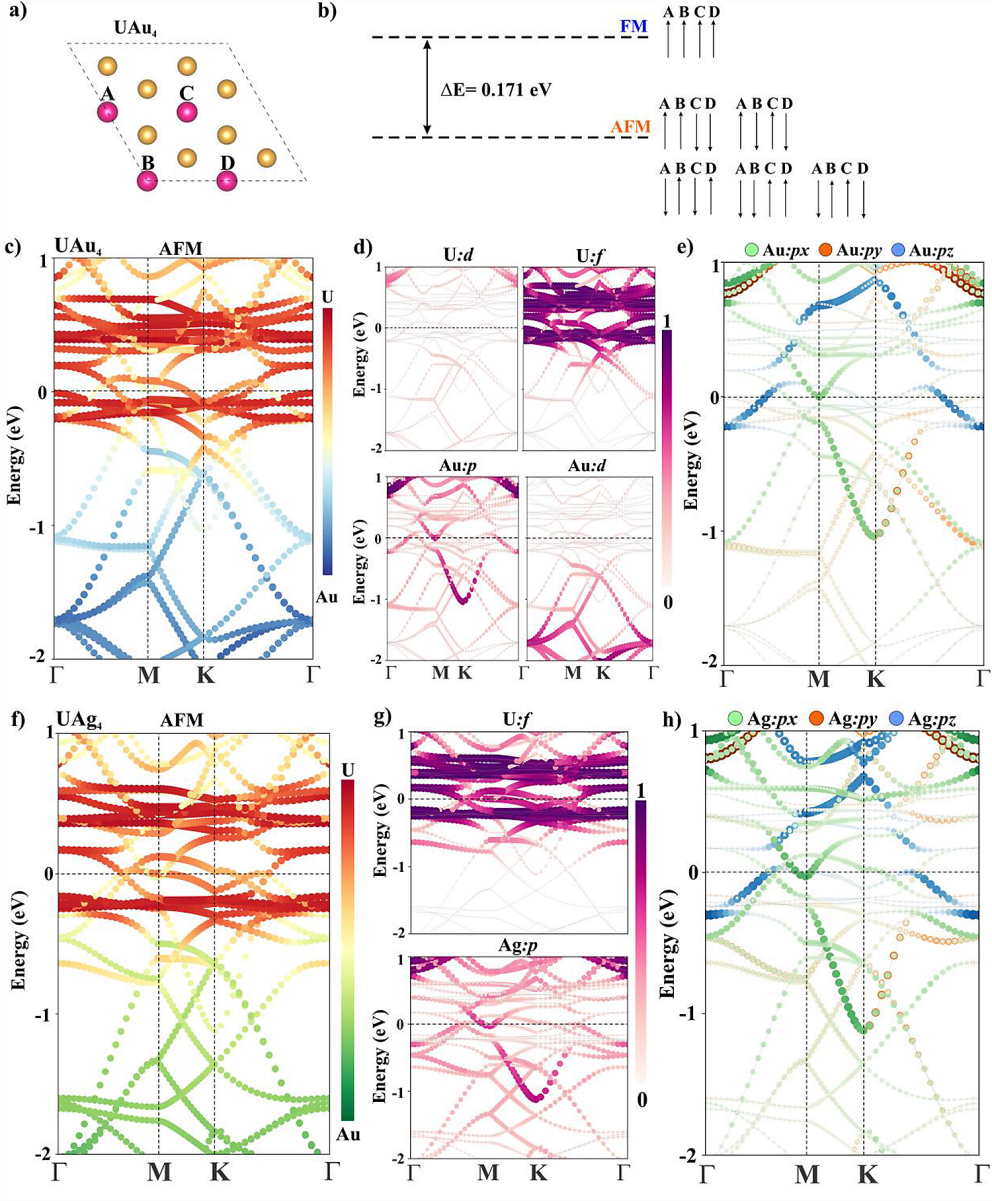
(a) 2 × 2 supercell of UAu_4_ monolayer; four uranium atoms are labeled as A/B/C/D, (b) comparative energy difference for different magnetic orientations; 5 configurations are considered for the AFM state as schematically presented, (c–e) atom/orbital projected band structure of the UAu_4_ supercell in the AFM configuration; Fermi level is set to zero, (f–h) atom/orbital projected band structure of the UAg_4_ supercell in the AFM configuration.

The energy differences between AFM and FM magnetic configurations are summarized in Table 3. Both spin-polarized and noncollinear DFT calculations suggest that all three monolayers, UCu_4_, UAg_4,_ and UAu_4,_ prefer antiferromagnetic configuration, as indicated by the negative value of the exchange energy. Among the three pristine monolayers, the magnitude of |Δ*E*_ex_| is the smallest for UCu_4_ and the largest for UAg_4_ which can be correlated to the size of Cu/Ag/Au. The exchange energy and *J* values are further calculated using meta-GGA and are listed in Table S6. We have also performed energy calculations considering different orientations of the magnetic moments for AFM as schematically shown in Figure 4a,b for the UAu_4_ monolayer. The AFM state is always found to be lower in energy by 0.171 eV than the FM state. The energy of all the five AFM configurations is found to be the same.

**Table 3:** Calculated Exchange Energy and Magnetic Coupling Constant Per Unit Cell

monolayers	Δ*E*_ex_ = *E*_AFM_– *E*_FM_ (meV)		*J* (meV/unit cell)	
	without SOC	with SOC	without SOC	with SOC
UCu_4_	–14.4	–33.02	–0.9	–2.06
UAg_4_	–202.19	–137.4	–12.63	–8.58
UAu_4_	–170.94	–109.68	–10.68	–6.85

### 3.4 Electronic Properties

The spin-polarized band structures in the AFM state are plotted in Figure 4 which indicates that the systems are metallic. The atom and orbital-projected band structures indicate that the bands near the Fermi level are mainly composed of U: f and Au: p (see Figure 4c,d) where the U: f states are found to be more dominant. The projected band structure depicts U: f and Au: p orbitals hybridizing near/above the Fermi level which provides an extra channel for indirect coupling between two neighboring U atoms through the Au-atom. Furthermore, all Au: p orbitals (p_*x*_, p_*y*_ & p_*z*_) are projected into the band structure in Figure 4e which shows a significant contribution of p_*x*_ and p_*z*_ but a slightly lower contribution of p_*y*_ in the energy window of [−0.5, 0.5] eV. These conduction electrons mediate indirect spin–spin coupling between U atoms through the Au atom. Similar electronic properties are observed for UAg_4_ ML (see Figure 4f–h).

### 3.5 Magnetic Anisotropy Energy

The magnetic anisotropy energies (MAE = *E*_⊥_ – *E*_∥_; *E*_⊥_ and *E*_∥_ represent total energies for the spin direction along the *z* and *xy* plane, respectively) of monolayers are calculated to be 8.28, 2.7, and 5.49 meV/atom for UCu_4_, UAg_4_, and UAu_4_ monolayers, respectively. The easy axis is found to be in-plane for all the monolayers. For comparison, the MAE of other 2D materials is 0.22 meV/atom for MnB and 0.48 meV/atom for FeB monolayers.^40^ The monolayers MnB and FeB have magnetic moments of 5μ_B_ and 3μ_B_, respectively. However, the origin of magnetism in those systems is from partially filled d-orbitals, whereas for 2D materials studied in this work, they are from partially filled f-orbitals which have higher energy.

### 3.6 Ground-State Magnetic Properties of Hydrogenated UX_4_ (X = Ag, Au) Monolayers

Since the ground state of UX_4_ monolayers is antiferromagnetic, we have examined if some strategy can make them ferromagnetic. We note that one of the earlier studies predicted that graphene can be ferromagnetic once it is semihydrogenated (called graphone).^41^ Verification of this prediction by later experiments^42^ has led to the use of hydrogen as a means to transform antiferromagnetic monolayers such as CrSe_2_, and CrTe_2_^43^ to a ferromagnetic state. These results demonstrated that hydrogenation leads to an expansion of the unit cell volume and drives the system toward ferromagnetic ordering as observed experimentally.^44^

**Figure 5:**
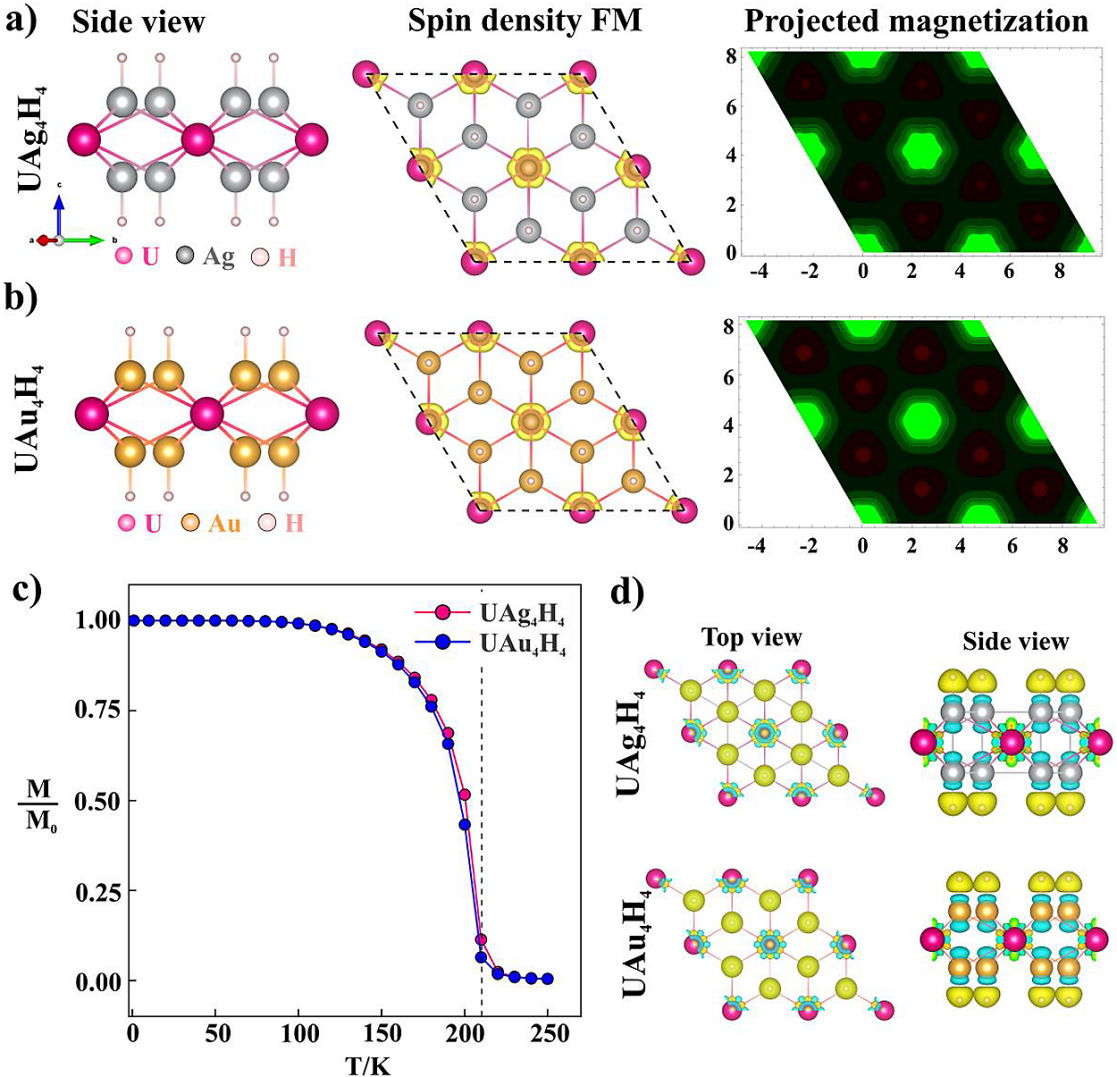
Geometrical side view, spin density in FM configuration and projected magnetization of spin density along the *z*-direction of (a) UAg_4_H_4_ and (b) UAu_4_H_4_ monolayer, (c) Variation of total magnetic moment per unit cell of fully hydrogenated UAg_4_ and UAu_4_ monolayers, and (d) top view of the 3D iso-surface (0.007 e/Å^3^) of the charge density difference; yellow and cyan color represent charge accumulation and depletion, respectively.

Here, we have examined if the hydrogenation of UAg_4_ and UAu_4_ monolayers could also lead to an antiferromagnetic to ferromagnetic transition. We focused on these two systems as the energy difference between the AFM and FM configurations is much larger than that of the UCu_4_ 2D monolayer. The geometrical view of the fully hydrogenated monolayers is presented in Figure 5a. A comparison of optimized lattice constants before and after hydrogenation is presented in Table 4. The lattice constants increase with hydrogenation, leading to an expansion of the unit cell by 2.7 and 3.14% for UAg_4_ and UAu_4_ monolayers, respectively. DFT calculation indicates that the total energy of the system is minimum when the H atom is adsorbed by the X atom compared to the U atom. The exchange energies of these hydrogenated monolayers are calculated using a 2 × 2 supercell for the FM and AFM configurations. The results are listed in Table S7. The antiferromagnetic coupling constant *J* decreases as the hydrogenation percentage increases and finally the magnetic ordering transforms from the antiferromagnetic to the ferromagnetic state. The spin density and projected magnetization along the *z*-direction of both UAg_4_H_4_ and UAu_4_H_4_ in FM configuration are plotted in Figure 5a,b. The Curie temperature that corresponds to the transition from the ferromagnetic to the paramagnetic phase is calculated using Monte Carlo (MC) simulations. The average magnetic moment at different temperatures has been evaluated based on the classical spin-half Ising model. In the MC simulations, a 30 × 30 supercell is used to calculate the Curie temperature. At each temperature, 10^5^ loops are taken to achieve an average magnetic moment value.

**Table 4:** Comparison of the Optimized Lattice Constants of 2D Materials with Varying Degrees of Hydrogenation

2D system	pristine	one side-hydrogenation	both side-hydrogenation and % expansion of unit cell
UAg_4_	4.742	4.867	4.87 (2.7%)
UAu_4_	4.704	4.847	4.852 (3.14%)

The Curie temperatures of UAg_4_H_4_ and UAu_4_H_4_ monolayers have been calculated using MC simulation; their variation of magnetization with temperature is provided in Figure 5c. The Curie temperatures of both monolayers are calculated to be ∼210 K, which is greater than the MnO_2_ monolayer^45^ (140 K) and comparable to the FeC_2_ monolayer^46^ (245 K), Cr@gtC_3_N_3_ (325 K),^47^ and MnB monolayer^48^ (345 K), ScCr_2_C_2_^49^ (187 K), Cr_2_O_3_ (185 K).^39^ Nearly the same value of the Curie temperature for both UAg_4_H_4_ and UAu_4_H_4_ monolayers is due to approximately the same lattice constant (see Table 4) and *J* value (see Table S7).

To further illustrate the phase transition from the AFM to the FM state with hydrogenation, we plot the deformation charge density, as shown in Figure 5d. The deformation charge density shows the accumulation of the electrons around the adsorbed H atom while electronic charge depletion from both the UAg_4_ and UAu_4_ layers suggests electron transfer from the UAg_4_/UAu_4_ layer to the H-atoms. Therefore, the adsorbed H atom reduces the electronic charge density from the UAg_4_/ UAu_4_ layer, mimicking the hole doping condition.^43,50^ The electron charge transfer per atom obtained from Bader charge analysis is listed in Table 5 and exhibits the same trend.

**Table 5:** Electronic Charge Transfer (e/atom) of U, X (Cu, Ag, Au), and H

	Δ*Q*_U_	Δ*Q*_X_	Δ*Q*_H_ (e/H)
UAg_4_	–1.4	+0.35	
UAg_4_H_2_	–1.29	+0.19	+0.43
UAg_4_H_4_	–1.39	–0.093	+0.442
UAu_4_	–1.957	+0.49	
UAu_4_H_2_	–2.037	+0.26	+0.49
UAu_4_H_4_	–1.88	–0.062	+0.53

For a systematic comparison, the effect of hydrogenation on the electronic properties of pristine and hydrogenated monolayers is studied and the corresponding DOS with and without hydrogenation are plotted in Figure S4. The higher energy electronic states in pristine monolayers are quenched with the addition of hydrogen which is consistent with the hole doping condition. In other words, the transport properties of conduction electrons are greatly affected by hydrogenation, and the antiferromagnetic to ferromagnetic transition under these circumstances provides a qualitative picture of the existence of an indirect exchange interaction between electron spins localized at the U-sites. Magnetism induced in these metallic systems due to f-orbitals of the U atom and the spin density localized at the U atom site can be attributed to the Ruderman–Kittle–Kasuya–Yosida (RKKY)-type indirect exchange interaction^51,52^ between neighboring electron spins through the conduction electrons.

**Figure 6:**
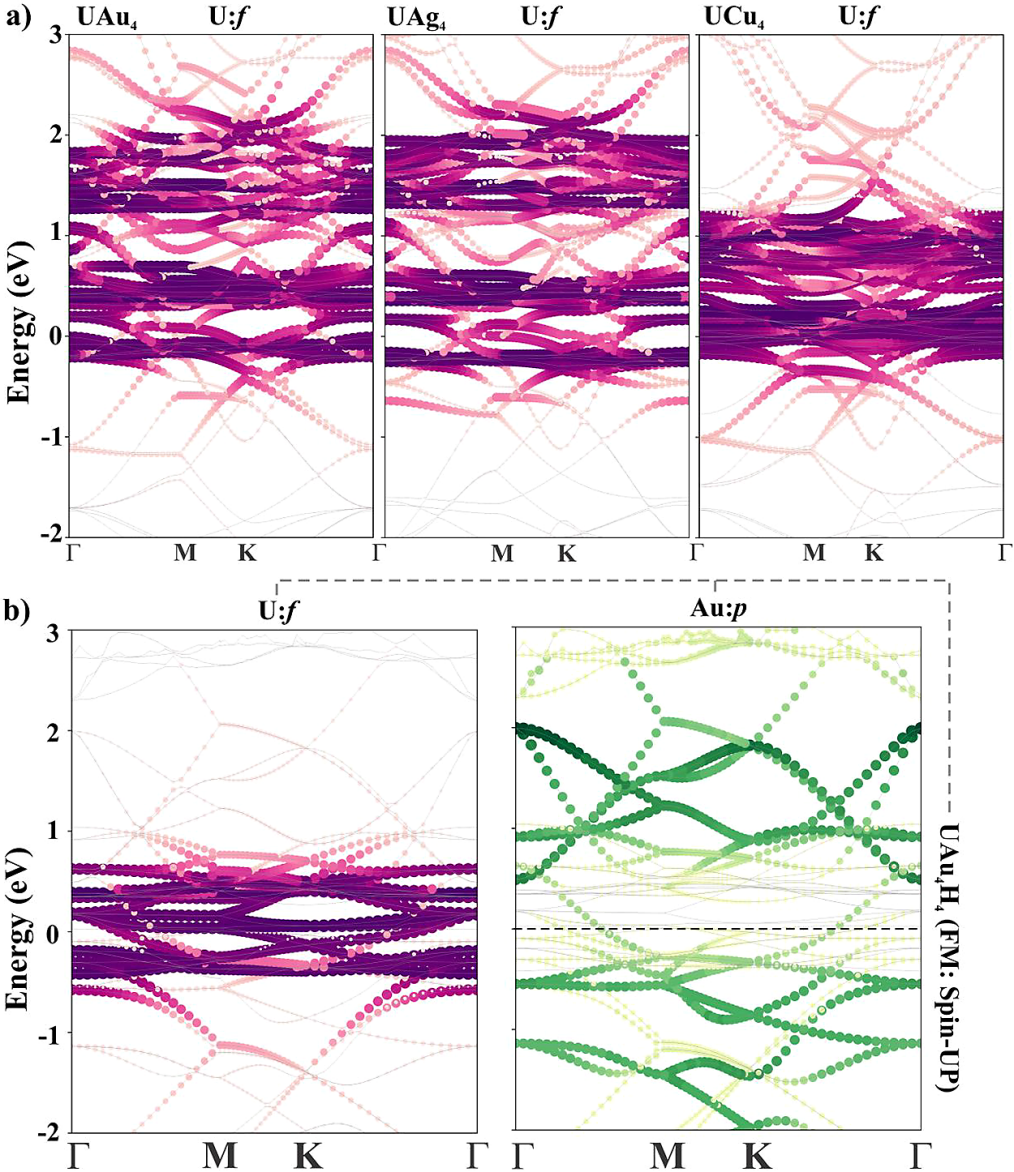
Orbital projected band structure of (a) pristine ML (AFM: spin-UP) and (b) hydrogenated ML (FM: spin-UP).

Further DFT calculations are performed to analyze the origin of phase transition and to confirm the type of magnetic exchange interaction. As hydrogen leads to an expansion of the lattice and magnetic transition, we wondered if the antiferromagnetic to ferromagnetic transition could also be induced by applying only biaxial strain to the pristine monolayers. To address this question, we studied the variation of the magnetic coupling constant *J* for UAg_4_ and UAu_4_ pristine monolayers under applied strain, , where *a*_0_ and *a* are the unstrained and strained lattice constants, respectively. The results are plotted in Figure S5 in the SI. Although it shows a clear variation, the *J* values are still negative even with a large biaxial strain of ∼10%. Furthermore, the magnetic coupling constants *J* calculated by removing the hydrogen atoms from hydrogenated UAg_4_ and UAu_4_ monolayer structure but maintaining the same expanded structure are found to be −15.65 meV/unit cell for the UAg_4_ monolayer and −9.99 meV/unit cell for the UAu_4_ monolayer. The negative value of *J* indicates that the AFM state is energetically preferred. These results confirm that the origin of the magnetic phase transition lies in the electronic interaction between adsorbed hydrogen atom and the UX_4_ monolayer i.e., electronic charge transfer from UAg_4_/UAu_4_ to hydrogen atom plays a decisive role in these phase transitions, not just an expansion of the unit cell. Hydrogenation lowers the number of conduction electrons of UAg_4_ and UAu_4_ monolayers as a clear difference can be observed in the DOS plot (see Figure S4) and from the data obtained in Bader charge analysis; charge deficiency is ∼1e and ∼2e on the U atom (see Table 5) in UAg_4_H_4_ and UAu_4_H_4_ monolayer, respectively. These results are consistent with the previous experiment on other materials.^53^ The surfaces of the 2D monolayers are Ag/Au atoms, which are noble metal atoms, and hence, they interact weakly with hydrogen. Nevertheless, we performed MD simulation at 300 K for 2 ps and present the results in Figure S6. The hydrogen atoms can be seen to remain near the surfaces. No significant distortion of the designed 2D structures is observed. The energy profile varies significantly in the hydrogenated UAu_4_ case because of the very weak interaction of H atoms with Au compared to that of Ag. Hence, instead of hybrid functionals or meta-GGA, we have calculated the energy exchange parameters using different *U*_eff_ values (*U*_eff_ = 3 eV) and listed the results in Table S8. The Δ*E*_ex_ and *J* values vary slightly.

For a better understanding of the AFM state in pristine and the FM state in hydrogenated MLs, U: f projected band structures are plotted in Figure 6a,b. For a systematic comparison, a slightly different energy window is considered. The higher energy U: f bands become localized as Au is substituted by Cu which provides a hint toward FM ordering as quantified by *J* values: UAu_4_ (|*J*| = 10.68 meV) > UCu_4_ (|*J*| = 0.9 meV). Furthermore, Figure 6b shows that when UAu_4_ ML is hydrogenated, U: f becomes more localized due to charge deficiency as mentioned earlier, and sandwiched between lower and higher energy Au: p bands. This will provide more channels for indirect coupling between the uranium atoms. The localization of U: f bands near the Fermi level gives a clear picture of the AFM to the FM transitions upon hydrogenation. In other words, in pristine U: f, bands hybridize with only higher energy Au: p bands whereas in hydrogenated MLs, U: f bands can interact with both higher and lower energy Au: p bands which leads to the AFM to the FM transition.

## 4 Conclusions

In summary, inspired by the recent experimental work on magnetic UAu_6_ cluster, calculations on a series of iso-structural and iso-electronic clusters composed of one uranium atom and six coinage metal atoms (UX_6_: X = Cu, Ag) have been carried out. The DFT calculations indicate that anionic UCu_6_^–^ and UAg_6_^–^ clusters have quartet spin multiplicity similar to that observed in the UAu_6_^–^ cluster. A magnetic moment of 3μ_B_ is found in these clusters, originating from the unoccupied f-orbitals of the uranium atom.

To see if the structure and magnetism of these clusters can be retained in a periodic crystal structure, we have designed a series of three atomic-thick UX_4_ (X = Cu, Ag, Au) monolayers. These structures are found to be thermodynamically and dynamically stable where the underlying cluster geometry as well as its magnetism is retained. However, two atomic-thick UX_2_ monolayers are found to be dynamically unstable. The ground state of all three monolayers is found to be antiferromagnetic as confirmed by spin-polarized calculations including spin–orbit coupling (SOC) and meta-GGA (SCAN). Although the interaction between hydrogen atoms and Ag/Au surfaces is weak, antiferromagnetic to ferromagnetic phase transition can be achieved via hydrogenation. The Curie temperatures of fully hydrogenated UAg_4_ and UAu_4_ monolayers are calculated using Monte Carlo simulation and found to be ∼210 K for both cases. From the optimized lattice constants of 2D monolayers with and without hydrogenation, we observe that hydrogenation leads to unit cell expansion, lowers the conduction electron density from the uranium atom, and drives the system toward ferromagnetic ordering. The long-range RKKY-type exchange interaction between neighboring spins is found to be dominant in these metallic systems. This is consistent with experimental observation in other systems. Most importantly, these results, inspired by the lessons from cluster science, show that new materials can be formed with clusters as building blocks. A recent experimental observation^32^ of nonfermi liquid behavior of bulk UAu_2_ structures below N*e*′el temperature provides a new platform to study the same in these strongly correlated 2D electron systems.

## Supporting Information Available

Electronic structure analysis of UCu_6_^–^ and UAg_6_^–^, geometrical view of U–Au bonds, variation of magnetic coupling constant (*J*) of pristine UAg_4_ and UAu_4_ monolayer under biaxial strain, DOS and projected DOS of pristine and hydrogenated UAg_4_ and UAu_4_ monolayers, AIMD simulation of (a) UAg_4_, (b) UAu_4_ monolayers with both sides hydrogenated, total energy of neutral and anionic clusters in Hartree unit having different spin states, NPA charge distribution and SCPA spin population of UX_6_^–^, orbital composition analysis of HOMO and LUMO with Ros-Schuit (SCPA) partition, atomic composition analysis of HOMO and LUMO, percentage orbital contribution to the magnetic moment, calculated exchange energy and magnetic coupling constant per unit cell with meta-GGA (SCAN), calculated exchange energy, magnetic coupling constant per unit cell, and Curie temperature obtained from the Monte Carlo simulation, and calculated exchange energy and magnetic coupling constant per unit cell with GGA-PBE with *U*_eff_ = 3 eV (PDF)
