# Supplementary material for: Magnetism of Otherwise Nonmagnetic Elements: From Clusters to Monolayers
Source: J Phys Chem C Nanomater Interfaces. 2024 Jul 16;128(29):12286–95. doi: 10.1021/acs.jpcc.4c03592 (PMC11284855; doi:10.1021/acs.jpcc.4c03592)
Supplement: Supplementary file 1 — jp4c03592_si_001.pdf [file jp4c03592_si_001.pdf]

# Supplementary Information (SI)

## Magnetism of Otherwise Nonmagnetic Elements: From Clusters and to Monolayers

Manish Kumar Mohanta and Puru Jena*

Department of Physics, Virginia Commonwealth University, Richmond, VA 23284, USA

*E-mail: pjena@vcu.edu

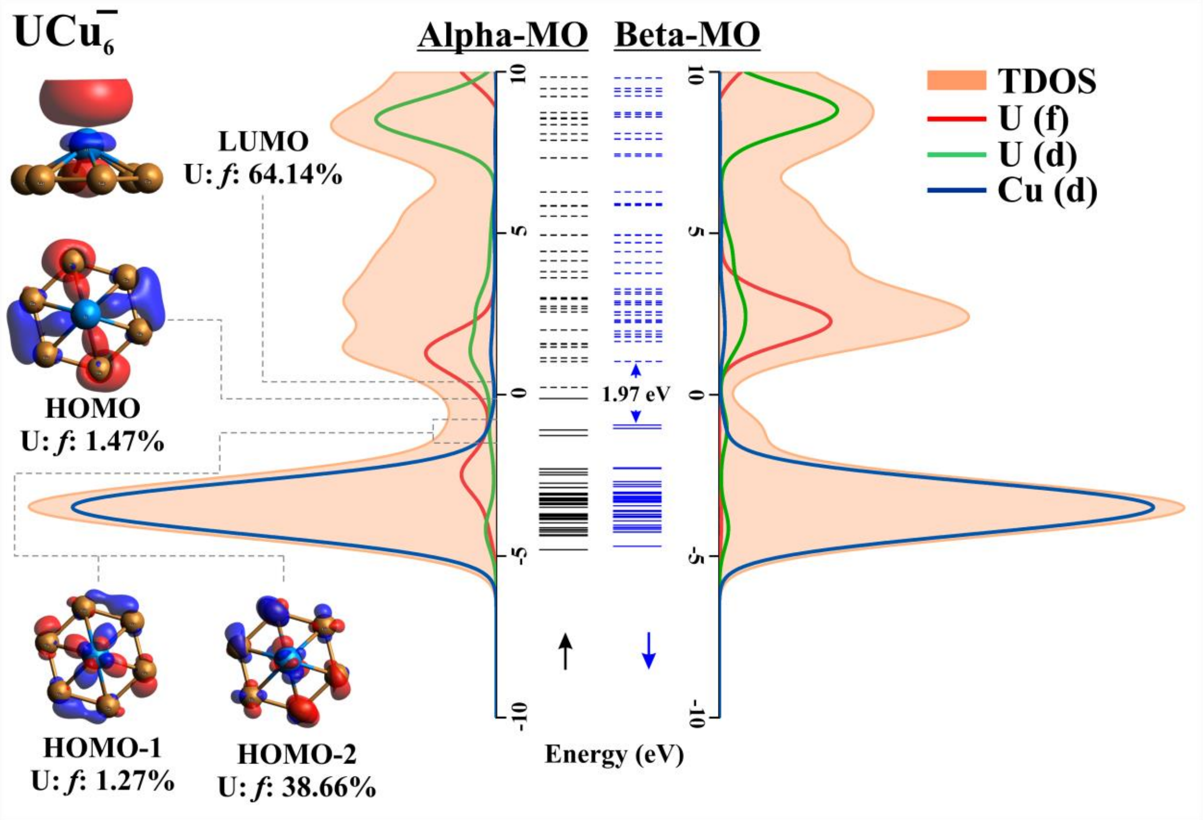


Figure S1 (a) Electronic structure analysis: partial density of states (pDOS) and selected α-orbitals of $UCu_6^-$; solid/dashed lines indicate occupied/unoccupied molecular orbital.

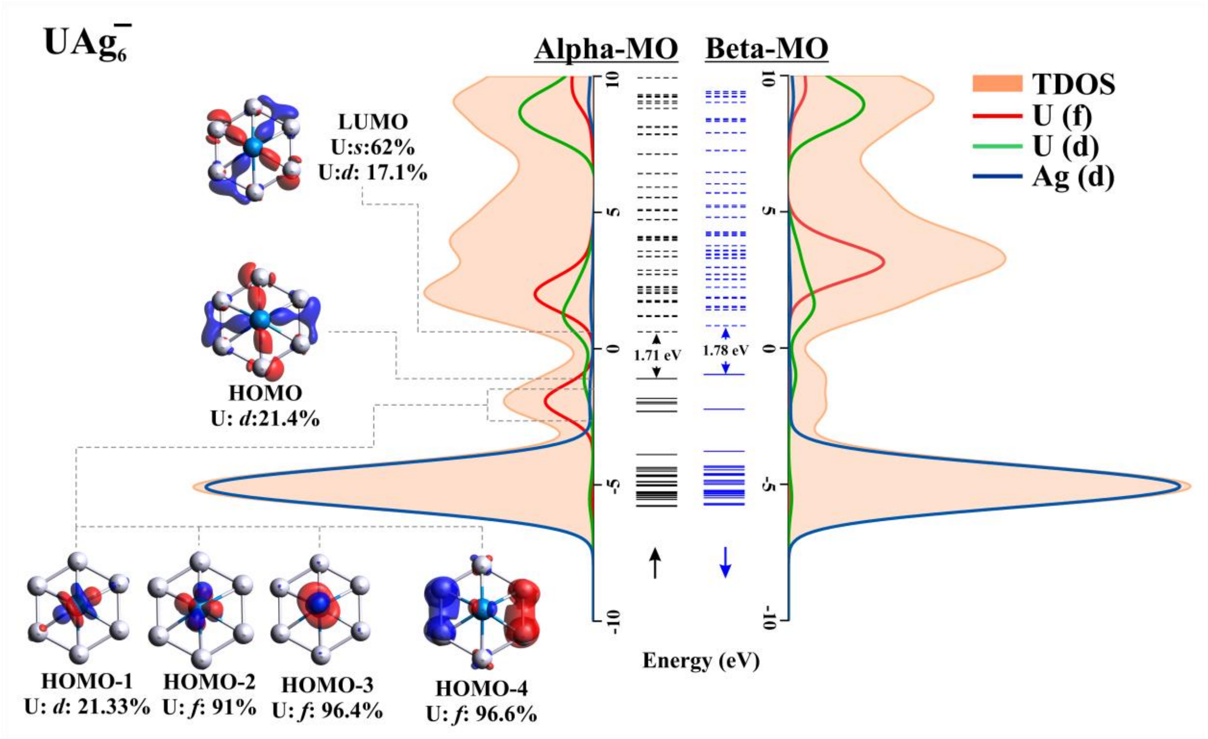


Figure S2 (a) Electronic structure analysis: partial density of states (pDOS) and selected α-orbitals of $UAg_6^-$; solid/dashed lines indicate occupied/unoccupied molecular orbital.

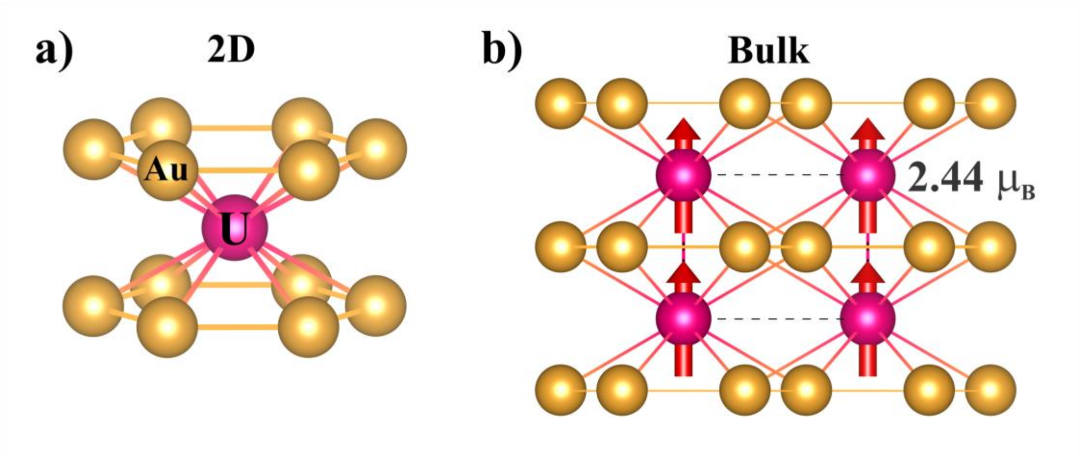


Figure S3 Geometrical view of (a) covalently bonded uranium atom with twelve Au-atoms in a periodic structure, (b) bulk unit cell with a magnetic moment at uranium site.

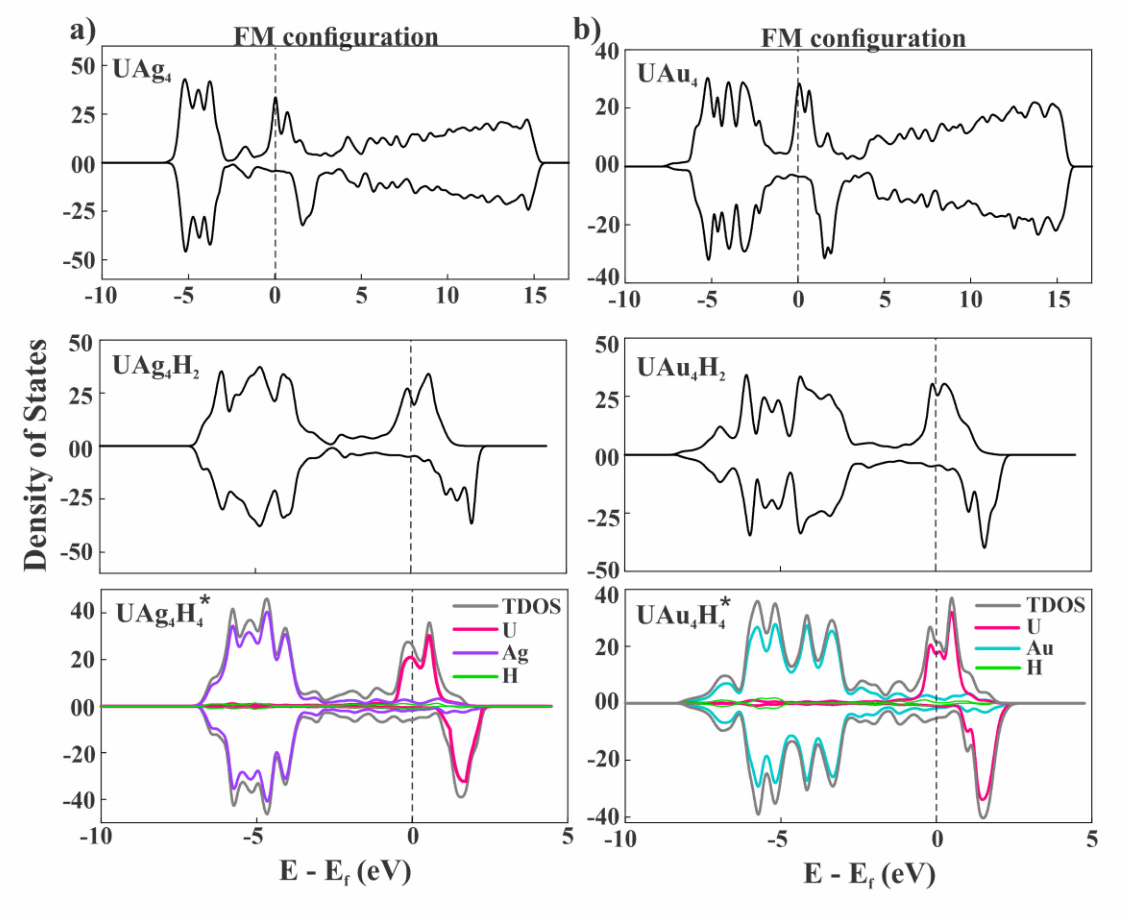


Figure S4 Calculated DOS and projected DOS of (a) pristine and hydrogenated $UAg_4$ and (b) $UAu_4$ monolayers; * indicates DOS plot of energetically minimum configuration state.

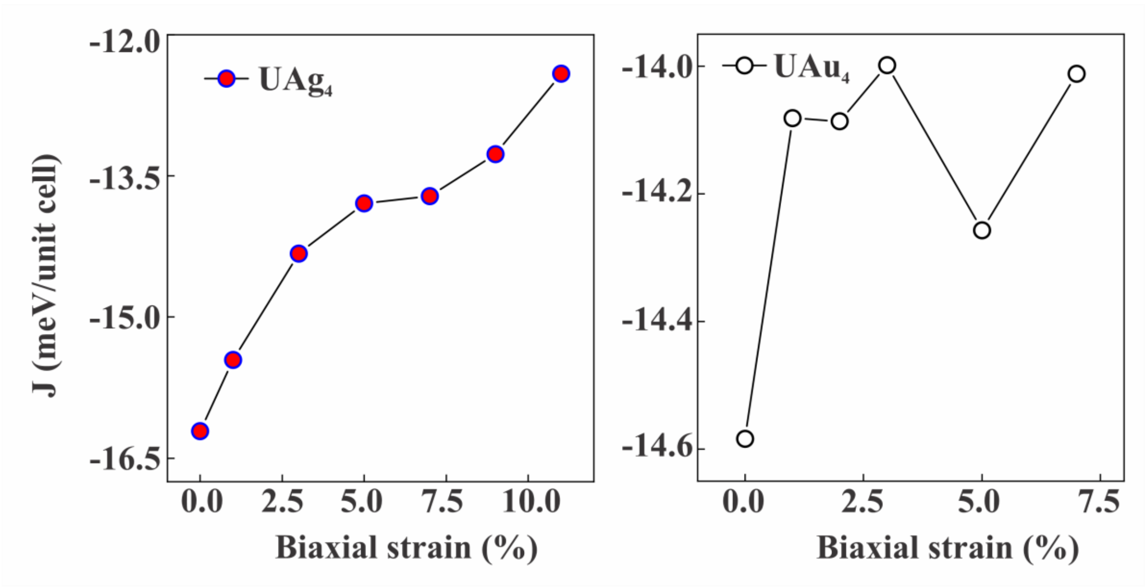


Figure S5 Variation of magnetic coupling constant of pristine $UAg_4$ and $UAu_4$ monolayer under biaxial strain.

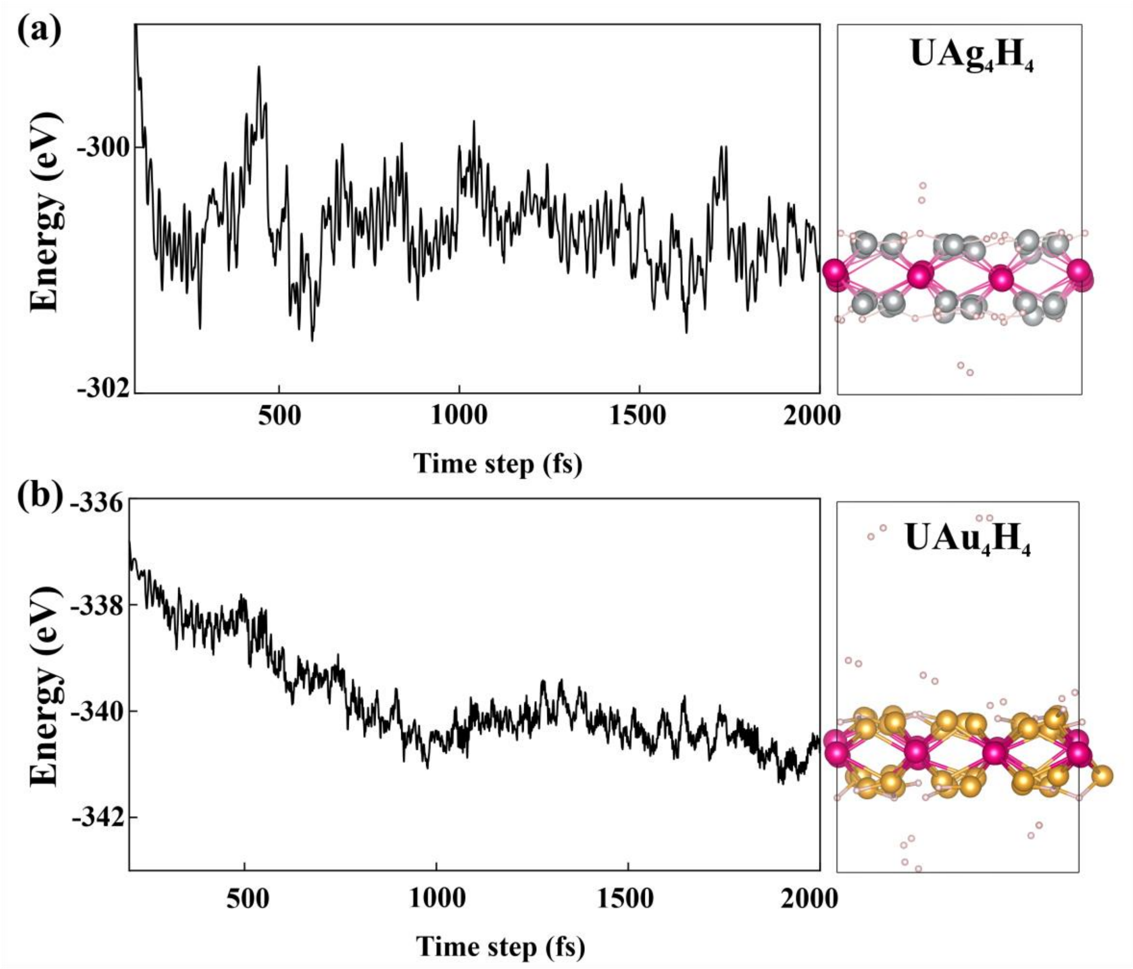


Figure S6 AIMD simulation of (a) $UAg_4$, (b) $UAu_4$ monolayers with both sides hydrogenated.

**Table S1 Total energy of neutral and anionic clusters in Hartree unit having different spin states**

| Cluster | singlet | triplet | quintet | Cluster | doublet | quartet | sextet |
|---|---|---|---|---|---|---|---|
| $UCu_6$ | -1658.8885 | -1658.9483 | **-1658.9592** | $UCu_6^-$ | -1658.990 | **-1659.0068** | -1659.005 |
| $UAg_6$ | -1356.7613 | -1356.8381 | **-1356.8441** | $UAg_6^-$ | -1356.8999 | **-1356.9253** | -1356.8967 |
| $UAu_6$ | -1289.6001 | -1289.6656 | **-1289.6682** | $UAu_6^-$ | -1289.7577 | **-1289.7809** | -1289.7240 |

| Table S2 NPA charge distribution and SCPA spin population of $UX_6^-$ | | | |
|---|---|---|---|
| No. | Atom | Spin population | NPA charge |
| $UAu_6^-$ | | | |
| 1 | Au | -0.02095839 | -0.19404768 |
| 2 | Au | -0.02472961 | -0.23506193 |
| 3 | Au | -0.02325670 | -0.21817658 |
| 4 | Au | -0.02328575 | -0.21820127 |
| 5 | Au | -0.02472637 | -0.23488676 |
| 6 | Au | -0.02098504 | -0.19394606 |
| 7 | U | 3.13794185 | 0.29432028 |
| Total net charge = -1 | | Total spin electrons = 3 | |
| | | | |
| $UAg_6^-$ | | | |
| 1 | Ag | -0.03367499 | -0.21239820 |
| 2 | Ag | -0.03323805 | -0.21028134 |
| 3 | Ag | -0.03501501 | -0.20502862 |
| 4 | Ag | -0.03496715 | -0.20440369 |
| 5 | Ag | -0.03327232 | -0.20973157 |
| 6 | Ag | -0.03357145 | -0.20952606 |
| 7 | U | 3.20373896 | 0.25136949 |
| Total net charge = -1 | | Total spin electrons = 3 | |
| | | | |
| $UCu_6^-$ | | | |
| 1 | Cu | -0.07227951 | -0.04528938 |
| 2 | Cu | -0.06741132 | -0.04370141 |
| 3 | Cu | -0.04727643 | -0.02331000 |
| 4 | Cu | -0.05575751 | -0.02286900 |
| 5 | Cu | -0.05754333 | -0.02284797 |
| 6 | Cu | -0.05705585 | -0.00937761 |
| 7 | U | 3.35732395 | -0.83260463 |
| Total net charge = -1 | | Total spin electrons = 3 | |

| Table S3 Orbital composition analysis of HOMO and LUMO with Ros-Schuit (SCPA) partition | | | | | | |
|---|---|---|---|---|---|---|
| Clusters | Spin | | *s* | *p* | *d* | *f* |
| $UCu_6^-$ | Alpha | HOMO | 52.26 | 31.9 | 14.33 | 1.478 |
| | | LUMO | 12.968 | 10.11 | 12.78 | 64.14 |
| | Beta | HOMO | 44.947 | 19.899 | 34.398 | 0.756 |
| | | LUMO | 43.81 | 41.5 | 14.49 | 0.19 |
| $UAg_6^-$ | Alpha | HOMO | 57.49 | 11.56 | 30.48 | 0.457 |
| | | LUMO | 62.92 | 18.55 | 18.12 | 0.397 |
| | Beta | HOMO | 63.661 | 13.775 | 22.39 | 0.174 |
| | | LUMO | 65.473 | 26.119 | 8.22 | 0.18 |
| $UAu_6^-$ | Alpha | HOMO | 0.214 | 0.683 | 4.843 | 94.26 |
| | | LUMO | 63.518 | 16.046 | 20.137 | 0.298 |
| | Beta | HOMO | 68.548 | 7.9 | 23.487 | 0.065 |
| | | LUMO | 67.166 | 22.916 | 9.777 | 0.141 |

| Table S4 Atomic composition analysis of HOMO and LUMO | | | | | |
|---|---|---|---|---|---|
| Cluster | Atoms | Alpha | | Beta | |
| | | HOMO | LUMO | HOMO | LUMO |
| $UCu_6^-$ | Cu | 16.985 | 25.87 | 83.65 | 31.15 |
| | U | 83.015 | 74.13 | 16.35 | 68.85 |
| $UAg_6^-$ | Ag | 78.14 | 23.86 | 85.22 | 28.55 |
| | U | 21.86 | 76.14 | 14.78 | 71.45 |
| $UAu_6^-$ | Au | 5.56 | 21.52 | 94.31 | 27.88 |
| | U | 94.44 | 78.479 | 5.69 | 72.112 |

| Table S5 Percentage orbital contribution to the magnetic moment | | | | | |
|---|---|---|---|---|---|
| Monolayers | Atoms | s | p | d | f |
| $UCu_4$ | Cu | / | 2.18 | 0.9 | / |
| | U | 0.8 | / | 3.86 | 92.2 |
| $UAg_4$ | Ag | / | 2.44 | / | / |
| | U | 0.87 | / | 3.47 | 93.15 |
| $UAu_4$ | Au | / | 1.91 | / | / |
| | U | 0.74 | / | 2.78 | 94.5 |

| **Table S6 Calculated exchange energy and magnetic coupling constant per unit cell with meta-GGA (SCAN)** | | |
|---|---|---|
| Monolayers | $\Delta E_{ex} = E_{AFM} - E_{FM}$ (meV) | J (meV/unit cell) |
| $UAg_4$ | -826.89 | -51.68 |
| $UAu_4$ | -231.91 | -14.49 |

| **Table S7 DFT calculated exchange energy, magnetic coupling constant per unit cell, and Curie temperature obtained from the Monte Carlo simulation** | | | |
|---|---|---|---|
| Monolayers | $\Delta E_{ex} = E_{AFM} - E_{FM}$ (meV) | J (meV/unit cell) | Curie temp (K) |
| $UAg_4H_2$ | -33.7 | -2.1 | / |
| $UAg_4H_4$ | 73.78 | 4.61 | 210 |
| $UAu_4H_2$ | -59.47 | -3.72 | / |
| $UAu_4H_4$ | 72.79 | 4.55 | 210 |

| **Table S8 Calculated exchange energy and magnetic coupling constant per unit cell with GGA-PBE with $U_{eff}$=3 eV** | | |
|---|---|---|
| Monolayers | $\Delta E_{ex} = E_{AFM} - E_{FM}$ (meV) | J (meV/unit cell) |
| $UAg_4H_4$ | 50.84 | 3.17 |
| $UAu_4H_4$ | 85.16 | 5.32 |
